# Segmental ischaemic infarction of the iris after autologous fat injection into the lower eyelid tissue: a case report

**DOI:** 10.1186/s12886-017-0599-8

**Published:** 2017-11-21

**Authors:** Jaeyoung Kim, Sa Kang Kim, Mee Kum Kim

**Affiliations:** 1 Laboratory of Ocular Regenerative Medicine and Immunology, Biomedical Research Institute, Seoul National University Hospital, 101 Daehak-ro, Jongno-gu, Seoul, 03080 South Korea; 2 Department of Ophthalmology, Seoul National University College of Medicine, 101 Daehak-ro, Jongno-gu, Seoul, 03080 South Korea

**Keywords:** Iris atrophy, Iris infarction, Iris depigmentation, Iris discoloration, Autologous fat injection, Fat embolism, Iris sphincter palsy, Case report

## Abstract

**Background:**

Autologous fat injection is getting popular in cosmetic procedures, however, still has a risk of fat embolism. Herein, we report the first case of segmental ischaemic infarction of the iris, which occurred after autologous fat injection into the lower eyelid.

**Case presentation:**

A 28-year-old Korean woman complained of the discolouration of the iris after the fat injection. Slit-lamp biomicroscopy revealed segmental depigmented atrophic iris with sectoral sphincter palsy.

**Conclusions:**

We found that iris atrophy could be caused by autologous fat transplantation. The plastic surgeons should pay more attention to possibility of fat embolism-induced ocular complications in the procedure of fat injection.

## Background

Facial injection procedures such as autologous fat or filler injections are becoming more common and acceptable worldwide. In comparison with other substances, autologous fat injection is regarded as safer because it uses material obtained from the patient’s own body. However, there are several case reports of critical complications due to fat embolism following autologous fat injection into the face including visual loss and cerebral infarction [1–3]. Herein, we report the first case of segmental ischaemic infarction of the iris which occurred after autologous fat injection into the lower eyelid.

## Case presentation

**Fig. 1:**
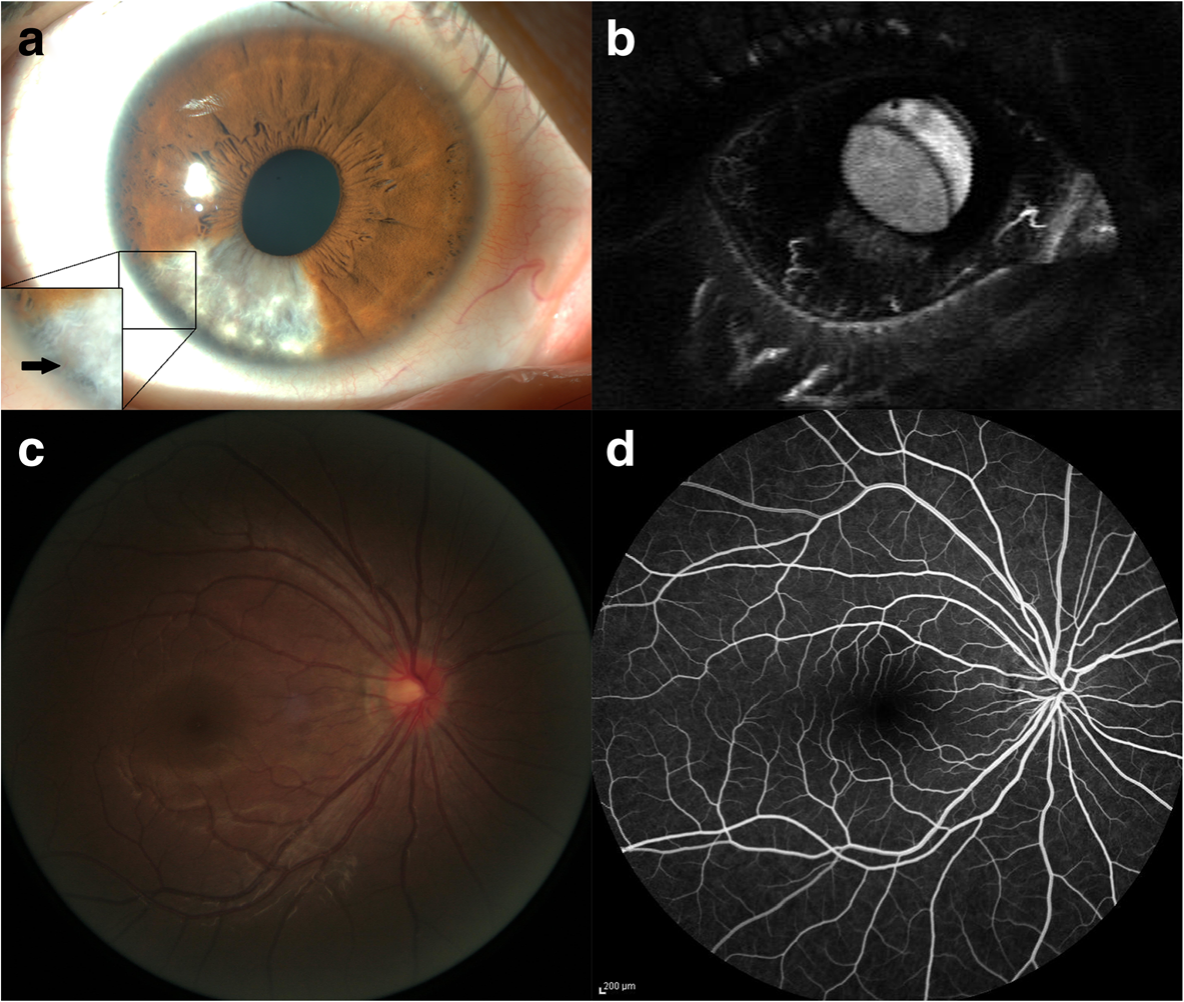
Anterior segment photography, showing segmental depigmentation of atrophic iris and new vessels (arrow) (**a**). Iris fluorescein angiogram showing focal hyperfluorescence of the decolorised area of the iris (**b**) [1]. Fundus photograph and fluorescein angiography of the right eye (**c** and **d**)

A 28-year-old woman complained of segmental iris depigmentation in the right eye, which she discovered 4.5 months after autologous fat injection into her face. The patient had no ocular medical or trauma history. She was in excellent general health, with no clinically relevant underlying diseases such as diabetes, hypertension or ischaemic disease. Her corrected visual acuity was 20/20, and her intraocular pressure was 18 mmHg. Slit-lamp biomicroscopy revealed a segmental whitish depigmented atrophic iris with new vessels between 6 and 8 o’clock and sectoral sphincter palsy of the decolourised iris (Fig. 1a). Fundus examination and fundus fluorescein angiography were normal (Fig. 1c and d). Iris fluorescein angiography showed focal hyperfluorescence of the decolourised area (Heidelberg Retina Angiograph (HRA), Heidelberg Engineering, Heidelberg, Germany) (Fig. 1b). Colour vision testing (Pseudoisochromatic plates, Richmond Products) and visual field testing (Goldmann perimetry) revealed no abnormalities. The iris lesion did not change during 2 months of follow-up.

## Discussion and Conclusions

Autologous fat transplantation-related vascular embolization has been recently reported. Acute vascular embolization of the ophthalmic or retinal artery can cause acute visual loss, while embolization of the cerebral artery can cause cerebral infarction [1–3].

Some causes of iris infarction have been reported. Kennedy has reported a case of sectorial iris infarction in an infant with meningococcal septicaemia [4]. In that case, meningococcal endotoxin-triggered disseminated intravascular coagulation was believed to be the cause of vaso-occlusion. It was previously reported that repeated iritis could be another cause of sectoral ischaemic necrosis of the iris [5]. Gupta presented a case of an elevated iris mass in a patient who had a history of recurrent herpetic keratoiritis. Sectoral iris biopsy revealed occluded vessels with massive pigment epithelial disruption and necrosis. In our case, the patient had no ocular history, and there was no sign of ocular inflammation or infection. Additionally, she was in good general health with no systemic diseases that could be associated with infarction. Therefore, autologous fat injection was considered to be the primary cause.

Blood supply to the orbit and the eye is from the ophthalmic artery, which has branches including the central retinal artery, lacrimal artery, ciliary arteries, and muscular branches. Because the facial area is rich in vasculature and has multiple vascular anastomoses, injected fat can be forced in a retrograde manner into one of the branches of the ophthalmic artery, especially under pressure [1]. In this case, after travelling back into the ophthalmic artery, the fat might have entered the long posterior ciliary artery (LPCA) or anterior ciliary artery (ACA) and passed through anastomoses of these arteries, which form the major arterial circle of the iris. It appeared that emboli lodged in one of the branches of the arterial circle of the iris to cause segmental ischaemic infarction [6]. As with our case, embolism of a minor artery such as the short posterior ciliary artery has been reported [7]. This complication was reported to have occurred immediately after an autologous fat injection. In our case, the iris abnormality was reported several months after fat transplantation. It is possible either that the atrophy may have occurred slowly due to multiple collateral vessels or she simply may not have noticed it until months later. The abnormality may have manifested late because of the abundant blood supply to the iris from the multiple anastomotic branches generated from the ACA and LPCA. Funk and Rohen reported that the ACA and LPCA are connected in all sectors [8]. Therefore, partial occlusion of the major arterial circle by an embolus obstructing one of them may have allowed the iris atrophy to progress slowly.

There have been no reports of iris infarction after autologous fat transplantation to date. In a patient who considers fat transplantation into the face for cosmetic purposes, the occurrence of recognizable iris discolouration is unlikely to be acceptable. Therefore, plastic surgeons should consider more closely the possibility of fat embolism-induced ocular complications resulting from fat injections.
